# Effects of endodontic irrigants on blood and blood-stained dentin

**DOI:** 10.1016/j.heliyon.2019.e01794

**Published:** 2019-05-23

**Authors:** Adrian Zollinger, Thomas Attin, Dirk Mohn, Matthias Zehnder

**Affiliations:** a Clinic of Preventive Dentistry, Periodontology and Cariology, University of Zurich, Center of Dental Medicine, Switzerland; b Institute for Chemical and Bioengineering, Department of Chemistry and Applied Biosciences, ETH Zurich, Switzerland

**Keywords:** Dentistry

## Abstract

**Objectives:**

This study aimed to investigate bleaching effects of common endodontic irrigants on human whole blood and blood-stained dentin. Specifically, it was assessed whether sodium hypochlorite at a clinically recommended concentration (2.5% NaOCl) would bleach with similar efficacy as a peroxide-based irrigant at higher molarity (5% H_2_O_2_). Furthermore, the effects of a NaOCl-compatible chelator with a high affinity to iron (Dual Rinse HEDP) were investigated.

**Methods:**

Human whole blood was mixed at a 1:20 ratio with either phosphate-buffered saline, 9% HEDP, 2.5% NaOCl, 2.5% NaOCl containing 9% HEDP, or 5% H_2_O_2_. Effects were assessed spectrometrically and photographically. Human dentin specimens were prepared with a methacrylate reservoir for liquids and a polished assessment side over 1 mm dentin thickness. Dentin was stained using human whole blood for 3 weeks and subsequently exposed to the irrigants for 60 min. Measurements were performed in the CIELAB color space. Results were compared using parametric tests with the alpha-type error set to 5%.

**Results:**

When directly exposed, the solutions containing NaOCl completely discolored the blood, while the 5% H_2_O_2_ exerted a bleaching effect without complete dissolution of dissolved matter, and the pure 9% HEDP had no effect at all. The NaOCl solutions bleached blood-stained dentin more efficiently than H_2_O_2_ (*p* < 0.05).

**Conclusions:**

Under the current conditions, the 2.5% NaOCl solution had a stronger bleaching effect on blood and blood-stained dentin than 5% H_2_O_2_. HEDP did not have any direct impact on blood color or NaOCl-derived bleaching.

## 1 Introduction

A history of dental trauma appears to be the most common reason for patients to have an individual tooth bleached [1]. Traumatized teeth can discolor because of pulp canal obliteration [2] or because of blood extravasation due to the rupture of blood vessels [3]. Bleeding with the subsequent diffusion of hemoglobin into the dentin appears to occur more frequently and result in stronger discoloration than mere pulp obliteration [1]. This discoloration originates from an accumulation of hemoglobin or other forms of hematin molecules in dentinal tubules [3]. The common way to whiten blood-stained teeth is the so-called walking bleach technique, which was introduced in 1963 [4]. Today, there are multiple products on the market to perform this treatment. They all are based on the sustained generation of hydroxyl radicals from a medication placed in the pulp chamber [5].

The topic of internal bleaching has been studied and reviewed extensively [6, 7]. However, one aspect appears to have been missed. A tooth with persistent blood-related staining of the dentin requires root canal treatment [8]. Consequently, the chemical agents that are used during that treatment may have an effect on discoloration. This has been suspected [9], but not studied. The most logical approach would be to use a hydrogen peroxide (H_2_O_2_) solution for root canal irrigation in such cases. Hydrogen peroxide solutions share their chemistry with the products used for non-vital tooth bleaching [7]. In endodontics, however, hydrogen peroxide solutions are not first choice, because they lack the ability to dissolve necrotic soft tissues [10] and are not the best disinfectants available [11, 12]. Nevertheless, H_2_O_2_ solutions are used for root canal irrigation [13], and have also been advocated to be applied alternatingly with sodium hypochlorite to create an effervescent cleaning effect [14]. Sodium hypochlorite (NaOCl) solutions are the most widely used irrigants [15] due to their unique effects on necrotic tissue and biofilms [12]. NaOCl is called “bleach” when referring to its use as a household chemical. NaOCl is a highly reactive, non-specific proteolytic agent that dissolves non-mineralized tissues [10], bacteria, and even the biofilm matrix [16]. It thus comes as no surprise that NaOCl also dissolves and, thus, discolors full blood within minutes [17]. Yet it is unclear what happens to the hemoglobin/protoporphyrin ring, though it is suspected that it decomposes under the influence of NaOCl. Furthermore, is not known how that effect compares to that of a peroxide-based irrigant.

Fe^2+^, i.e. iron in the ferrous state, is the central ion in each of the four heme complexes contained in hemoglobin. Chelating agents are used in endodontic irrigants to remove the smear layer and condition the dentin for the root filling procedure [18]. In theory, these could also have an effect on hemoglobin via the chelation of the iron. Among the chelators that are currently used in endodontic irrigants, 1-hydroxyethane 1,1-diphosphonic acid (HEDP) appears to form the strongest chelate complexes with iron [19]. In addition, HEDP can be combined with NaOCl to form an “all-in-one” irrigant with concurrent deproteinizing and demineralizing properties [20].

It was the goal of the current study to compare the effects of H_2_O_2,_ NaOCl and a combination of NaOCl with HEDP in aqueous solution on human whole blood from a blood donation and blood-stained human dentin in vitro.

## 2 Materials and methods

### 2.1 Human materials

The human whole blood used in this study was excess material used for blood agar plates in microbiology. It contained citrate phosphate dextrose as an anticoagulant. In addition, it contained a stabilizer consisting of sodium chloride, adenine, glucose monohydrate, and mannitol [21].

The teeth in this study were caries-free third molars extracted for reasons not related to the current work. All patients gave informed written consent that these teeth could be used to do anonymized in-vitro tests. None of these human-derived materials could be traced back to their donors, the current protocol under local law was exempt from the necessity to obtain an individual ethics approval [22].

### 2.2 Root canal irrigants

The root canal irrigants used in the current study were 2.5% NaOCl, phosphate-buffered saline (PBS, Oxoid, Hampshire, England), 9% HEDP in water or 2.5% NaOCl, and 5% H_2_O_2_. The NaOCl (PanReac Applichem, Darmstadt, Germany) and H_2_O_2_ (Sigma Aldrich, St. Louis, MO, USA) solutions were obtained by diluting more concentrated stock solutions (10% NaOCl and 50% H_2_O_2_) in deionized water, and assessed using iodometric titration [23]. To obtain a 9% HEDP solution, 9.1 g of deionized water was mixed with 0.90 g of etidronate powder (Dual Rinse, Medcem, Weinfelden, Switzerland) as described [24]. To get a corresponding mixture containing 2.5% NaOCl and 9% HEDP, 9.1 g of 2.75% NaOCl (obtained from the stock solution described above) was mixed with 0.90 g of etidronate powder. NaOCl/HEDP mixtures were prepared immediately prior to the experiments, as they are not storable [24].All the concentrations reported in this communication relate to weight/total weight of the solutions under investigation. The pH of these solutions was measured using a calibrated pH electrode (827 pH lab; Deutsche Metrohm, Filderstadt, Germany).

### 2.3 Effects of irrigants on human whole blood

Absorption patterns of the irrigants and their combinations with whole blood were collected using a monochromatic ultra-violet (UV) spectrometer (U 2010, Hitachi, Tokyo, Japan) as follows: 10 mL of test or control solutions were transferred into 15-mL polypropylene centrifugation tubes (Corning, NY, USA). These pure irrigants were used for blank measurements by transferring 2 times 3 mL from the Corning tube to a polystyrene cuvette of 10 × 10 × 45 mm (Sarstedt AG, Sevelen, Switzerland). Fifty μL of human whole blood were added to the remaining 4 mL of irrigants in the Corning tube, which was then rotated in the vertical plane for 2.5 min at 10 rpm (Stuart Rotator SB3, Core-Palmer, Stone, UK). Because of the high amounts of nascent oxygen in the 5% H_2_O_2_ solution with the admixed blood, all irrigant/blood combinations were degassed in vacuum in a borosilcate beaker for 2.5 min. Subsequently, 3 mL of the mixture was transferred to a polystyrene cuvette and the spectrophotometry pattern was obtained. These experiments were performed in triplicates.

All irrigant/blood reactions described above were also monitored in glass petri dishes and filmed using a digital microscope (Dino-Lite, AnMo Electronics, Hsinchu, Taiwan) at 20× magnification.

### 2.4 Standardized dentin specimens

**Fig. 1:**
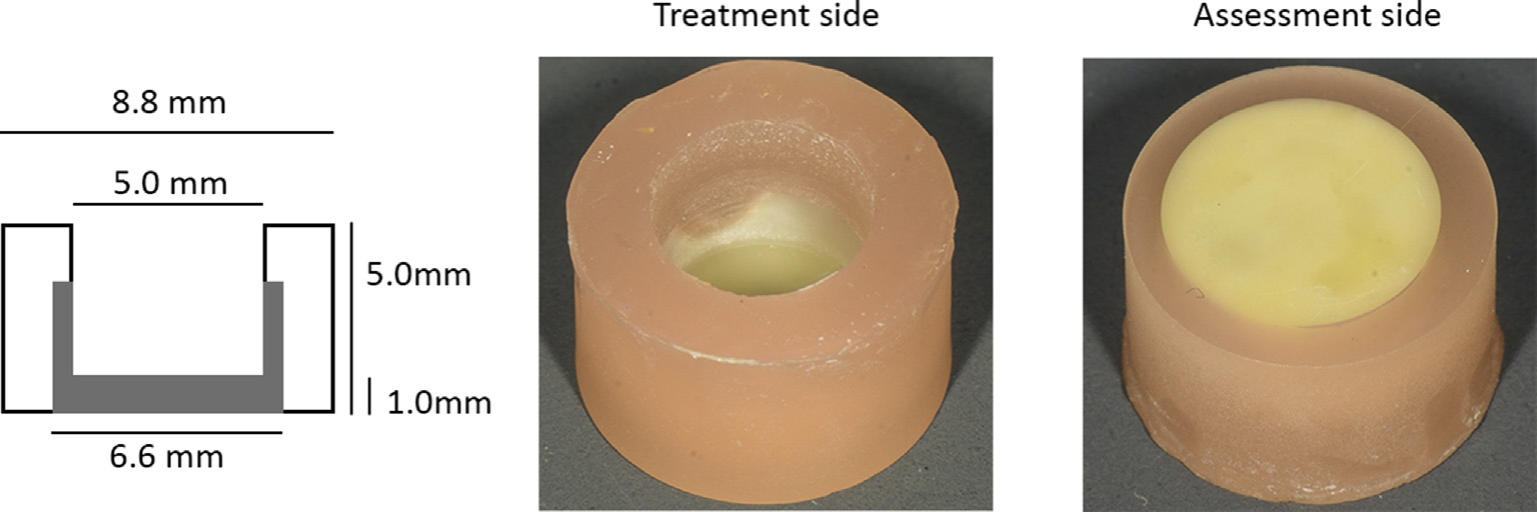
Technical drawing (left panel) and representative photographs of the model used in this study. Human dentin from the roof of the pulp chamber of extracted sound third molars was embedded in methacrylate. A reservoir for the blood and the subsequent treatment (Treatment side, middle panel) was drilled from the pulpal side of the dentin, whilst the coronal aspect was polished for color assessment (Assessment side, right panel).

Third molars were embedded with their apical root aspects in methacrylate (Paladur, Kulzer, Hanau, Germany) on an SEM stub (Wenka, Karl Wenger SA, Courgenay, Switzerland). Using a low-speed sawing apparatus (IsoMetTM LS, Buehler, Esslingen, Germany) equipped with a diamond-coated wheel saw (Struers, Ballerup, Denmark), each tooth was horizontally sectioned at the cemento-enamel junction under water cooling. Subsequently, a cylinder of 6.6 mm diameter was drilled in the coronal dentin in length axis of the tooth using a water-cooled diamond core drill in a customized apparatus (Proxxon, Trier, Germany). A second horizontal cut was then made using the low speed saw to obtain a dentin cylinder 6.6 mm in diameter and 3.5 mm in height. This dentin cylinder was again embedded in methacrylate (Paladur) to form a cylinder of 8.8 mm diameter with the coronal dentin surface exposed. This surface was polished (Tegramin 30, Struers) using silicon carbide grinding paper (Prüfag, Schlieren, Switzerland) of 2500 and then 4000 Grit. Subsequently, a hollow cylinder with a diameter of 5 mm was prepared in the middle of each specimen using a water-cooled diamond core drill, so that the dentin had a remaining thickness of 1 mm and the lateral walls of the resulting holding chamber a height of 4 mm (Fig. 1). Each specimen was immersed in 17% EDTA (Kantonsapotheke, Zürich, Switzerland) for 30 s and then washed in distilled water. An indication line was drawn on the outside of each specimen using a permanent marker, so that the specimen could be positioned on the spectrophotometer in a standardized manner (see below).

### 2.5 Spectrophotometry

Color changes on the polished dentin surfaces (Fig. 1, assessment side) were assessed using a black/white-calibrated spectrophotometer (Konica Minolta CM-2600d, Tokyo, Japan) connected to an external computer running the analysis software (Spectra Magic NX, version 2.8, Konica Minolta). Specimens were positioned on the device using a customized aluminum holder so that they could be reassessed in the exact same position. Measurements were performed in the CIELAB color space in reflectance mode, where the L* value indicates the white to black, a* the green to red, and b* the blue to yellow hue. Images were taken with a field of view of 3 mm (Target Mask A147, Konica Minolta) under simulated natural light illumination (D65). Raw data were used for further analysis. Measurements were performed in triplicates at baseline, after blood staining, and at different time points during irrigation (see below).

### 2.6 Blood staining and irrigation

To stain the dentin specimens, 50 μL of human whole blood was pipetted into the holding chamber (Fig. 1), and the specimens were centrifuged for 2 min at 1200 x g (Hermle Z320, Gosheim, Germany) in 15-mL Corning tubes with their polished dentin surface (assessment side) facing down. Subsequently, remaining blood was removed from the holding chamber. The specimens were then stored in 100% humidity for 3 weeks at 37 °C in an incubator (NCU-Line IL 23, VWR International, Leuven, Belgium). As assessed in a pilot study over 10 weeks, the color stabilized after 3 weeks under the conditions described here.

To irrigate the specimens, 50 μL of irrigating solution was pipetted into the holding chamber. Irrigants were replaced, i.e. removed and 50 μL of fresh solution applied, every 10 min. Spectrophotometric measurements were performed after 10 min, 30 min, and 60 min of storage at room temperature (25 °C). The pure 9% HEDP solution was excluded from these experiments, as it was realized that it did not have an impact on blood color. Before the measurements, the irrigant was removed and refreshed thereafter. A timer was used so that the irrigation times were exactly the same for all specimens. Experiments were repeated 7 times (n = 8) on separate days. In each run, one specimen per group (PBS, 2.5% NaOCl, 2.5% NaOCl +9% HEDP, and 5% H_2_O_2_) was assessed to avoid bias.

### 2.7 Data presentation and analysis

Effects of the irrigants under investigation on human whole blood are presented qualitatively. Data related to the color difference of the blood-stained dentin are shown as CIELAB ΔE* (change in overall color) and ΔL (bleaching effect) values. The effects of irrigant and time as categorical independent variables on ΔE* and ΔL values (continuous dependent variables) was tested using two-way analysis of variance (ANOVA). Values between different irrigants at a given point in time were analyzed using one-way ANOVA followed by Tukey's HSD test. The alpha-type error was set to 5% (p < 0.05).

## 3 Results

**Fig. 2:**
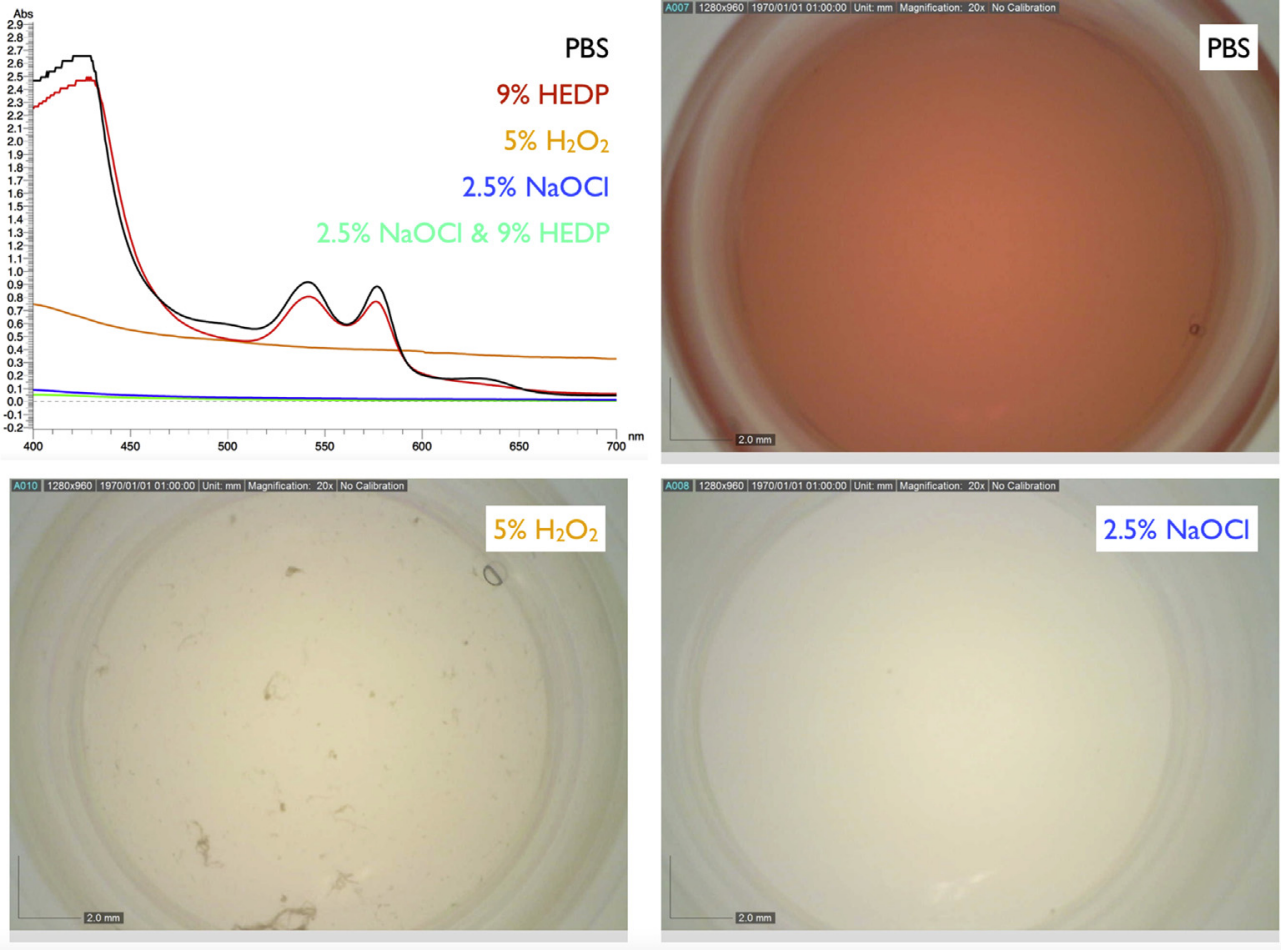
Top left: Absorption patterns of human whole blood mixed with different irrigants (1:80 mixtures, 5 min). The typical double peak of oxygenated hemoglobin can be seen in mixtures of full blood and PBS as well as HEDP, indicating that the latter did not affect the iron in the hem that binds the oxygen. Photographic images show the typical red color of blood mixed with PBS (top right), the bleaching effect of H_2_O_2_ with denatured protein remnants (bottom left), and the complete dissolution of all visible blood constituents by NaOCl (bottom right).

The pH of the solutions used in this study was 7.3 for the PBS, 12.3 for the 2.5% NaOCl, 12.1 for the fresh mixture containing 2.5% NaOCl and 9% HEDP, 11.2 for the pure 9% HEDP, and 4.5 for the 5% H_2_O_2._ Light absorption spectra of the 1:80 mixtures of human whole blood with PBS revealed the typical twin peaks related to oxygenated hemoglobin at 540-2 nm and 576-8 nm (Fig. 2, top left). The same peaks were observed when the blood was mixed with the pure 9% HEDP solution, indicating a continued presence of the iron in the heme molecule. In contrast, the peaks were absent in the corresponding mixture with 5% H_2_O_2_. Some light-absorbing matter, however, remained in the 5% H_2_O_2_ solution. In contrast, the irrigants containing NaOCl completely cleared the blood (Fig. 2, top left). The HEDP contained in the NaOCl solution did not hamper its bleaching effect. This was corroborated by photographic images obtained using a digital microscope (Fig. 2). After 5 min, the 5% H_2_O_2_ solution had bleached the blood, but some apparently denatured protein agglomerates remained, and the solution was not entirely clear. In contrast, the 2.5% NaOCl solutions had dissolved all blood components at that time.

**Fig. 3:**
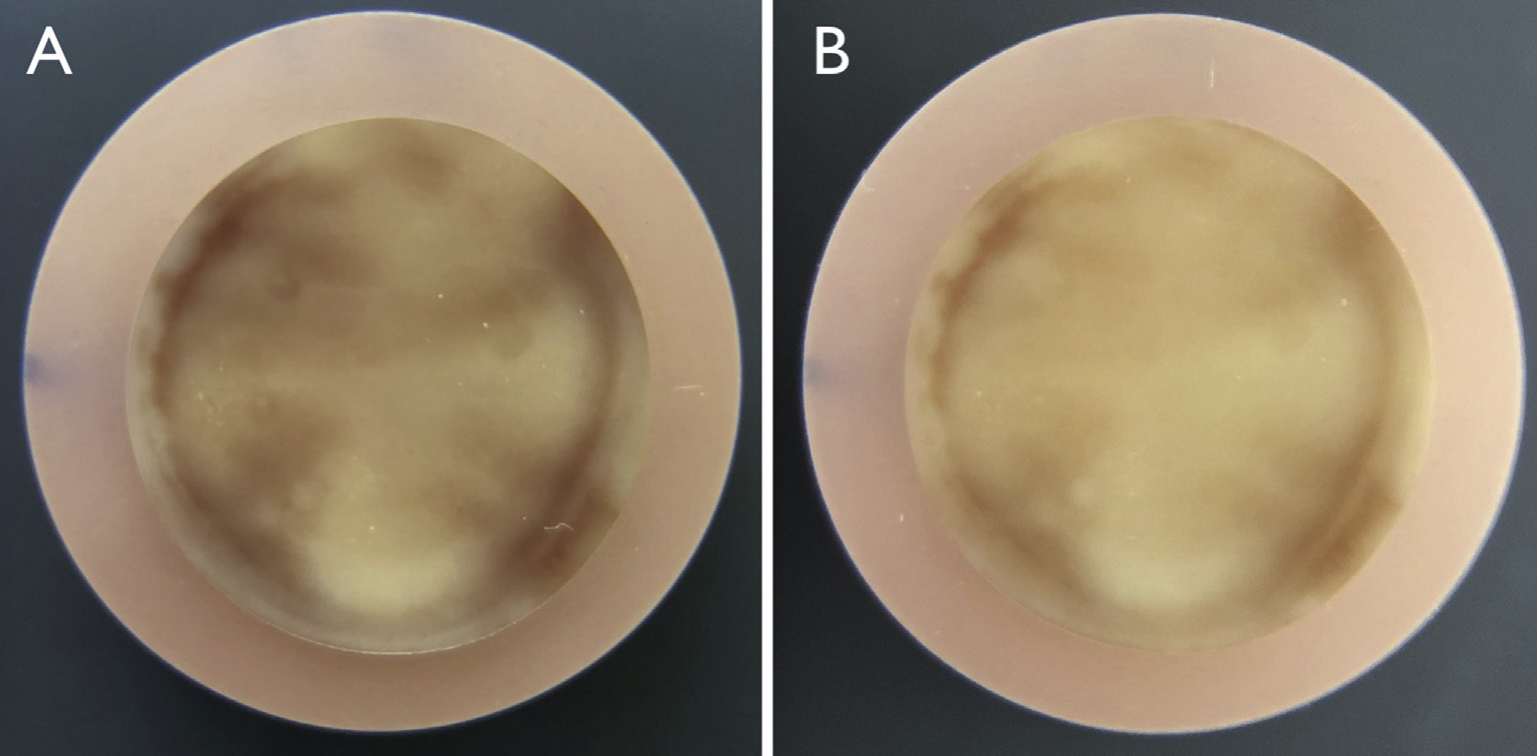
Representative photographic images of a human dentin specimen stained with human whole blood for 3 weeks (A) and then bleached from the treatment side (see Fig. 1) for 60 min using a combined aqueous irrigant containing 2.5% NaOCl and 9% HEDP. The ΔE* value in this specific specimen was 4.7, the ΔL was 3.2.

Experiments performed with the blood-stained human dentin specimens exposed to the irrigants under investigation showed that statistically significant overall color (Table 1) and lightness (Table 2) changes occurred after 10 min and increased over time. Two-way ANOVA revealed that the type of irrigant and time both significantly (*p* < 0.05) affected ΔE* and ΔL values. Effects on color change and bleaching caused by the 2.5% NaOCl solutions (with and without HEDP) were roughly double those of the 5% H_2_O_2_ at all times (*p* < 0.05 after 30 min and 60 min). These color changes were discernible by eye, yet did not reflect a reconstitution of the dentin to its appearance before blood staining (Fig. 3).

**Table 1:** Color changes (ΔE* values) of blood-stained dentin caused by irrigants under investigation over time (means and standard deviations, n = 8).

Irrigant	10 min	30 min	60 min
PBS	1.9 ± 1.2^A^	2.4 ± 1.0^A^	3.0 ± 1.3^A^
2.5% NaOCl	2.8 ± 0.8^A,B^	4.4 ± 0.8^B^	6.2 ± 2.7^B^
2.5% NaOCl +9% HEDP	3.7 ± 2.2^B^	4.9 ± 2.4^B^	5.8 ± 2.2^B^
5% H_2_O_2_	1.4 ± 0.7^A^	1.9 ± 1.0^A^	3.1 ± 1.4^A^

Identical superscript letters indicate that there was no significant difference at the 5% level between two irrigants at that point in time (ANOVA, Tukey's HSD).

**Table 2:** Bleaching effect (ΔL values) on blood-stained dentin caused by irrigants under investigation over time (means and standard deviations, n = 8).

Irrigant	10 min	30 min	60 min
PBS	−1.4 ± 1.1^A^	−2.0 ± 1.1^A^	−2.7 ± 1.4^A^
2.5% NaOCl	1.9 ± 1.3^B,C^	3.2 ± 1.4^C^	4.9 ± 1.7^C^
2.5% NaOCl +9% HEDP	2.9 ± 1.6^C^	4.2 ± 1.8^C^	5.1 ± 2.2^C^
5% H_2_O_2_	0.9 ± 0.7^B^	1.4 ± 0.6^B^	2.3 ± 1.0^B^

Identical superscript letters indicate that there was no significant difference at the 5% level between two irrigants at that point in time (ANOVA, Tukey's HSD).

## 4 Discussion

The current study showed that endodontic irrigating solutions containing NaOCl have a stronger bleaching effect on blood and blood-stained dentin than counterparts containing H_2_O_2_. HEDP, a chelator with a strong affinity to iron, did not have any direct impact on blood color, yet did not impair the bleaching effect of NaOCl under current conditions.

The concentrations of the irrigants used in this study were those recommended for clinical usage [12]. H_2_O_2_ and NaOCl are both oxidizing agents used for bleaching. However, they were present at two different pH regions: H_2_O_2_ was acidic (pH 4.5) and NaOCl was alkaline (pH 12). Peroxide bleaching should have a stronger oxidation potential but its optimal working area is at higher pH (>10.5) where it dissociates into H^+^ and HO_2_^-^. The perhydroxyl ion HO_2_^-^ is responsible for bleaching and especially stain removal and it is more present at higher pH [25]. It further decomposes into hydroxyl ions OH^−^, which aid to solubilize stains or precipitates. In general, sodium percarbonate, sodium perborate or calcium peroxide are substances, which are often used for bleaching in household products with a corresponding liquid, forming an environment with a high pH and, thus, a much stronger bleaching effect. The here applied NaOCl solutions have a high pH and contain predominantly the oxidizing hypochlorite ion OCl^−^. Despite the fact that its reduction potential is smaller, the bleaching effect under current conditions was more intense compared to that of hydrogen peroxide. The presence of the strong oxidizing agent (OCl^−^) and a high concentration of hydroxyl ions, representing the ideal combination for stain removal, resulted in the increased bleaching effect for NaOCl. This might explain NaOCl's unique dissolving effect on organic matter, too [26]. The current results are in line with observations on connective tissue [10] and agarose [16], where NaOCl had a dissolving effect while H_2_O_2_ did not. The double peaks at 540-2 nm and 576-8 nm in the light absorption spectra of the mixture between whole blood and the 9% HEDP solution clearly relate to oxyhemoglobin [27]. This and the finding that the corresponding mixture with PBS showed the same peaks strongly suggests that HEDP did not remove the iron from the heme.

The blood-stained dentin model used in the current study has never been used as such. Other groups studying bleaching effects on blood-stained dentine used enamel-dentin disks from bovine incisors [28, 29] or intact human premolars [30, 31] to assess the effects of commercially available bleaching gels used in the walking bleach technique. In the current study, however, irrigants, i.e. liquids, were used. Consequently, a model was created that featured a standardized reservoir for application of the irrigants, as well as a standardized dentin diameter and thickness. This model neither reflects the spatial environment of the human pulp space nor the irrigant exchange that occurs during a root canal treatment. Nevertheless, results were comparable to clinics: oxidizing irrigating solutions, especially NaOCl, bleached blood stains in dentin. However, that effect per se was not sufficient to return the dentin to its natural color, and a walking bleach procedure would still be necessary. In the current model, PBS caused a change in ΔE* values. This was most likely due to a darkening of the blood-stained dentin (Table 2) by rewetting. As a further limitation, the current model that did not involve infection of the dentin, which may be present in blood-discolored traumatized teeth *in vivo*. However, infected teeth may be easier to bleach than non-infected counterparts [32], because the protoporphyrin ring of the heme is more likely to remain intact in the absence of bacterial invasion [3].

The set-up that was used for the assessment of color changes (Konica Minolta CM-2600d, Program: Spectra Magic NX, version 2.8, Konica Minolta) can quantify ΔE*ab (CIE 1976), ΔE00 (CIE DE2000) and each component of lightness, saturation and hue. We decided to show results as ΔE* and ΔL (bleaching effect) values, because that is the standard in dentistry that everybody understands [33]. However, it may be so that ΔE00 values detect more subtle changes and may be closer to human perception [34].

The current finding that hypochlorite has a higher efficacy than peroxide on blood stained dentin opens the question why products using this chemistry are not available for the home-bleaching process. Indeed, chlorinated lime (calcium hypochlorite) was used in earlier days to bleach teeth [6]. In essence, calcium hypochlorite is similar to sodium hypochlorite in its pH and effects [35], with the difference that it is available as a stable salt and could thus also be used as a suspension with controlled release of its bleaching component. This, however, would not be good for the mechanical integrity of the tooth, as the hypochlorite affects the collagen in the root dentin with increasing concentration and time of exposure [36, 37].

Future studies could look at the effects of endodontic irrigants on other types of dentin stains such as those caused by amalgam [38], tetracycline [39], or the Bi contained as a radiopacifier in many materials [40].

## Declarations

### Author contribution statement

Adrian Zollinger: Conceived and designed the experiments; Performed the experiments; Wrote the paper.

Thomas Attin, Matthias Zehnder: Conceived and designed the experiments; Analyzed and interpreted the data; Wrote the paper.

Dirk Mohn: Conceived and designed the experiments; Analyzed and interpreted the data; Contributed reagents, materials, analysis tools or data; Wrote the paper.

### Funding statement

This research did not receive any specific grant from funding agencies in the public, commercial, or not-for-profit sectors.

### Competing interest statement

Dirk Mohn and Matthias Zehnder report a conflict of interest in that he has applied for a patent related to the use of phosphonate salts in endodontics (EP3284456A1; US 20180042821).The other authors deny any conflict of interest related to this work.

### Additional Information

No additional information is available for this paper.
